# The Therapeutic Mechanisms of Huayu Quban Capsule in Treating Acne Vulgaris Are Uncovered Through Network Pharmacology and Molecular Docking

**DOI:** 10.1111/jocd.16632

**Published:** 2024-11-17

**Authors:** Lei Zhang, Yu Huang, Wei Zhu

**Affiliations:** ^1^ Kunshan Hospital of Traditional Chinese Medicine Kunshan China

**Keywords:** acne vulgaris, compound traditional Chinese medicine, Huayu Quban capsule, molecular docking, network pharmacology

## Abstract

**Purpose:**

To uncover how the Huayu Quban (HYQB) capsule treats acne vulgaris (AV) through the use of network pharmacology and molecular docking technology.

**Methods:**

The traditional Chinese medicine system pharmacology database (TCMSP) was used to identify the components and potential targets of HYQB capsule. Targets related to AV were identified by screening the GeneCards, Disease Gene Network (DisGeNET) and Online Mendelian Inheritance in Man (OMIM) databases. The protein–protein interaction (PPI) network between targets of active ingredients and AV targets was built using the STRING database. Cytoscape3.7.2 software was used to create the visualization network for the ‘herb‐component‐target’ and identify the key targets. Gene ontology (GO) and Kyoto Encyclopedia of Genes and Genomes (KEGG) were utilized for functional enrichment analysis of the primary targets. Subsequently, molecular docking technology was employed to confirm the interaction between key components and core targets.

**Results:**

The technique discovered 50 different active substances and 270 associated therapeutic targets in the HYQB capsule as well as predicting 70 targets for treating acne vulgaris. Cytoscape hubba plug‐in identified 19 key target genes, with the top 5 being TNF, IL1B, CCL2, SIRT1, IFNG, and IL10. Analysis of KEGG pathways revealed significant enrichment of immune‐related pathways, including TNF and IL‐17 signaling pathways, among the target genes. The HYQB capsule also involves lipid and atherosclerosis, Th17 cell differentiation, and the AGE‐RAGE signaling pathway in diabetic complication signaling pathways. Molecular docking results showed that quercetin, luteolin, kaempferol, and wogonin, the core components of HYQB, had good binding ability with the first 4 core targets.

**Conclusions:**

The HYQB capsule may have a synergistic effect on inhibiting sebaceous adipogenesis and sebum cell differentiation and play an effect on AV through anti‐inflammatory and antioxidant effects of different signaling pathways.

Acne vulgaris (AV), a prevalent inflammatory skin condition, impacts individuals of all races and is most prevalent in the 15–20 age group. Affecting around 85% of young people, this skin disease is the most prevalent [1]. The burden of this condition, along with its clinical and psychological consequences, is significant. The way acne looks and its after effects, like scars and changes in skin color, can often cause emotional and social distress [2]. The development of acne is complicated, with various internal and external factors affecting oil‐producing glands that play a role in the formation of acne spots, particularly anaerobic bacteria and inflammation [3, 4]. While there are many treatments available for acne, there is no definitive evidence of effective treatment methods, such as oral isotretinoin [5], which can have psychiatric side effects and often lead to the acne coming back [6]. Hence, there is an urgent requirement for a novel treatment option for this stubborn skin condition.

The Huayu Quban (HYQB) capsule is a recently developed traditional Chinese medicine product that was included in the 2020 version of the Chinese Pharmacopeia. The complete formula consists of Radix Bupleuri, *Mentha spicata* L., *Scutellariae radix*, Angelicae Sinensis Radix, *Carthamus tinctorius*, and *Radix paeoniae*. It is mainly used to treat facial skin diseases such as melasma, acne, and rosacea. Combining these medications can help alleviate wind and heat, improve blood flow, and reduce blood stasis [7]. Clinical experience has shown that Huayu Quban capsule is particularly beneficial for patients with acne vulgaris characterized by wind‐heat and blood stasis. However, the scientific basis and underlying pharmacological mechanisms of HYQB remain unclear and require further research.

The complexity of Chinese medicine's active components means that most Chinese medicines have therapeutic effects that involve multiple targets and pathways in the human body. The traditional model of ‘one drug, one target, and one disease’ is not sufficient to fully capture the comprehensive and systematic nature of Chinese medicine [8]. Network pharmacology, a combination of systems biology and pharmacology, is a new and effective approach for drug development and understanding potential therapeutic mechanisms [9]. This study analyzed the potential mechanism of HYQB against AV by creating a network pharmacological model and establishing a molecular docking model between central targets and active substances, offering a fresh perspective on HYQB's mechanism [10]. See Figure 1 for a detailed explanation of the process.

**FIGURE 1:**
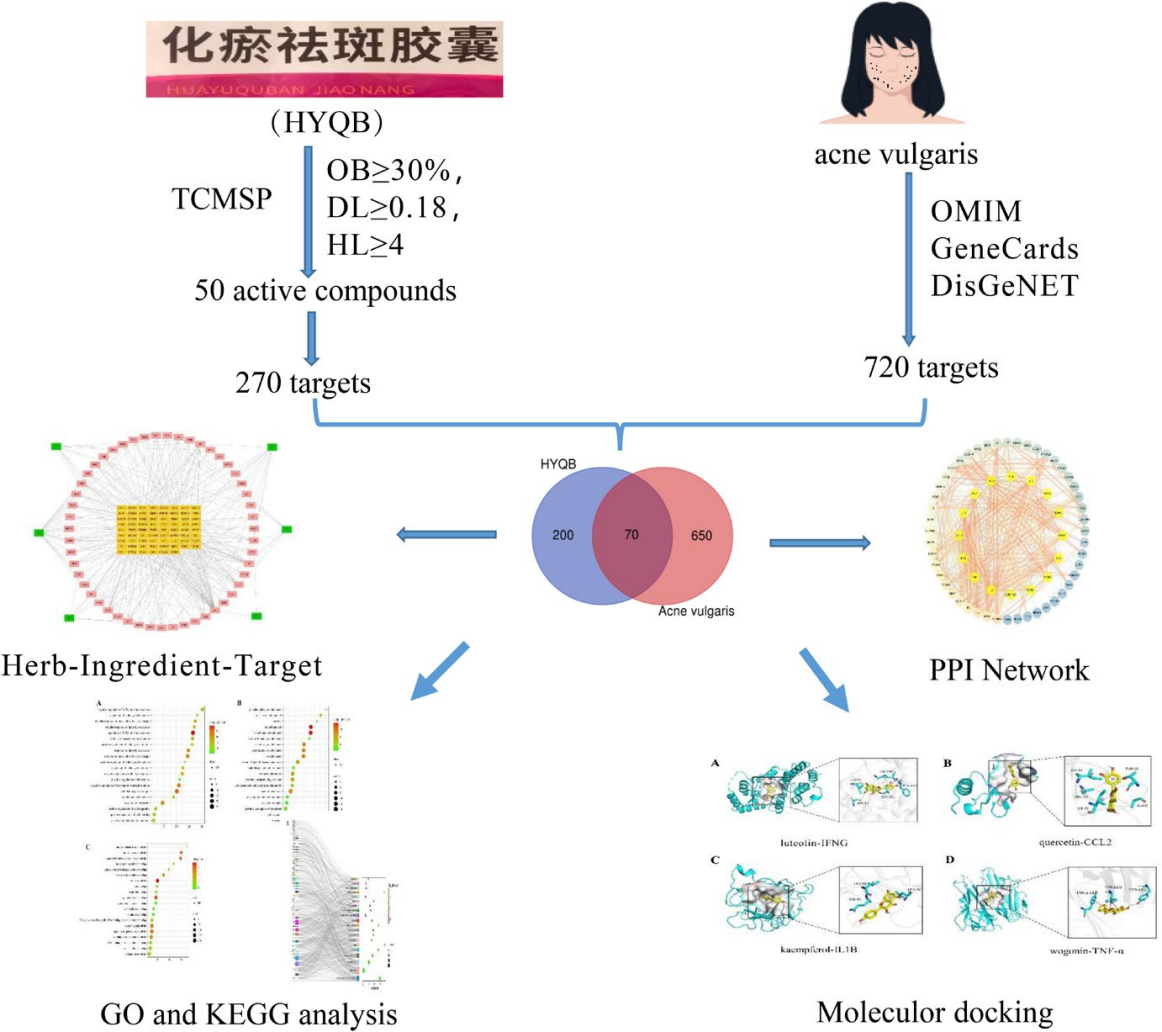
The unique structure for analyzing the network pharmacology of HYQB in the treatment of acne vulgaris.

## 1 Materials and Methods

### 1.1 Collection of Active Compounds and Potential Targets of HYQB Capsules

The bioactive ingredients of six different herbs in HYQB capsules were obtained from the analysis platform TCMSP (https //old.tcmsp‐e.com/molecule.php). The critical parameters for absorption, distribution, metabolism, and excretion (ADME) modeling were established as OB ≥ 30%, DL ≥ 0.18, and HL ≥ 4. The mol2 format files of these active compounds were then downloaded for future molecular docking [11, 12]. The target's official name is sourced from UniProt (http //www.UniProt.org/), specifying the species as “Homo sapiens.”

### 1.2 Screening Potential Targets for the AV Treatment With the HYQB Capsule

AV‐related targets were collected from OMIM (https://www.omim.org/), DisGeNET (https://www.disgenet.org/), and GeneCards (https://www.genecards.org/) databases [13]. After integrating all target genes, removing duplicates, we compiled a comprehensive list of associated targets [14].

### 1.3 Network Construction of Common Targets Between Active Compounds and AV

The potential target genes of HYQB in the treatment of AV were obtained from Bioinformatics and Systems Biology (https://bioinformatics.psb.ugent.be/webtools/Venn/). Then, Cytoscape3.7.2 was used to construct a visual network to reflect the relationship between HYQB, active components, and common targets, so as to obtain the information of the core compounds.

### 1.4 PPI Network Construction of Potential Therapeutic Targets

The shared objectives were uploaded to the STRING 12.0 database and used to build a PPI network for “Homo sapiens” with a minimum confidence score of 0.7 [15]. After eliminating the unconnected nodes from the protein–protein interaction network, the data in TSV format is imported into Cytoscape3.7.2 for visualization. Next, the CytoNCA tool in Cytoscape software is utilized to examine the network topology parameters of the target, which encompass Degree Centrality (DC), Betweenness Centrality (BC), and Closeness Centrality (CC). HYQB capsules focused on central targets with degree values higher than the average values (DC > 10, BC > 0.007, CC > 0.45) [16], and the top 4 were chosen for molecular docking.

### 1.5 Analysis of Enrichment in GO and KEGG Pathways

Metascape (https://metascape.org/) was used to conduct GO enrichment and KEGG pathway enrichment analysis on the shared targets of HYQB and AV [17]. The GO analysis included biological process (BP), cellular component (CC), and molecular function (MF), with the top 20 entries for each component examined based on a *p*‐value threshold of 0.05.The KEGG analysis was conducted to label the signaling pathways related to these targets, and the top 20 entries were chosen with a *p*‐value below 0.05.

### 1.6 Molecular Docking Verification

We utilized the 3D structures of the top 4 core targets identified through PPI network analysis to perform molecular docking using Autodock software (http://autodock.scripps.edu/) [18]. The binding energy between the active ingredient and protein was calculated using AutodockVina (http://vina.scripps.edu/), and the results were visualized with Pymol and LigPlot [19].

## 2 Results

### 2.1 Selection of HYQB Capsule‐Related Compounds and Potential Targets

Nighty nine compounds of HYQB were obtained from TCMSP, of which 50 compounds met the parametric conditions of ADME (Table 1). Afterwards, we determined the possible targets of every part of HYQB, resulting in a total of 270 targets once duplicates were eliminated (Table S1). While the number of targets associated with each herb in HYQB varies, there are common targets shared among several herbs. The findings indicate that various components of HYQB have intricate and varied regulatory effects on similar targets [20].

**TABLE 1:** Informations of active ingredients of the HYQB capsule.

No.	Molecule ID	Molecule name	OB (%)	DL	HL
1.	MOL001645	Linoleyl acetate	42.1	0.20	7.48
2.	MOL000354	Isorhamnetin	49.6	0.31	14.34
3.	MOL004718	α‐spinasterol	42.98	0.76	6.46
4.	MOL004609	Areapillin	48.96	0.41	16.52
5.	MOL004598	3,5,6,7‐tetramethoxy‐2‐(3,4,5‐trimethoxyphenyl) chromone	31.97	0.59	15.54
6.	MOL013187	Cubebin	57.13	0.64	12.4
7.	MOL000449	Stigmasterol	43.83	0.76	5.57
8.	MOL000422	Kaempferol	41.88	0.24	14.74
9.	MOL000098	Quercetin	46.43	0.28	14.4
10.	MOL001689	Acacetin	34.97	0.24	17.25
11.	MOL000359	Sitosterol	36.91	0.75	5.37
12.	MOL000006	Luteolin	36.16	0.25	15.94
13.	MOL000358	Beta‐sitosterol	36.91	0.75	5.36
14.	MOL002714	Baicalein	33.52	0.21	16.25
15.	MOL002881	Diosmetin	31.14	0.27	16.34
16.	MOL005573	Genkwanin	37.13	0.24	16.10
17.	MOL004328	Naringenin	59.29	0.21	16.98
18.	MOL000471	Aloe‐emodin	83.38	0.24	31.49
19.	MOL005190	Eriodictyol	71.79	0.24	15.81
20.	MOL004355	Spinasterol	42.98	0.76	5.32
21.	MOL006992	(2R,3R)‐4‐methoxyl‐distylin	59.98	0.30	15.08
22.	MOL001924	Paeoniflorin	53.87	0.79	13.88
23.	MOL002773	Beta‐carotene	37.18	0.58	4.36
24.	MOL000953	CLR	37.87	0.68	4.52
25.	MOL002712	6‐Hydroxykaempferol	62.13	0.27	14.29
26.	MOL002721	Quercetagetin	45.01	0.31	13.82
27.	MOL002695	Lignan	43.32	0.65	14.88
28.	MOL000552	5,2’‐Dihydroxy‐6,7,8‐trimethoxyflavone	31.71	0.35	16.47
29.	MOL000173	Wogonin	30.68	0.23	17.75
30.	MOL002909	5,7,2,5‐tetrahydroxy‐8,6‐dimethoxyflavone	33.82	0.45	15.94
31.	MOL002915	Salvigenin	49.07	0.33	15.87
32.	MOL002917	5,2′,6′‐Trihydroxy‐7,8‐dimethoxyflavone	45.05	0.33	16.37
33.	MOL002927	Skullcapflavone II	69.51	0.44	16.14
34.	MOL002928	Oroxylin a	41.37	0.23	17.15
35.	MOL002932	Panicolin	76.26	0.29	16.78
36.	MOL002933	5,7,4′‐Trihydroxy‐8‐methoxyflavone	36.56	0.27	16.93
37.	MOL002934	NEOBAICALEIN	104.34	0.44	16.50
38.	MOL000525	Norwogonin	39.40	0.21	16.93
39.	MOL001458	Coptisine	30.67	0.86	9.33
40.	MOL002897	Epiberberine	43.09	0.78	6.1
41.	MOL008206	Moslosooflavone	44.09	0.25	17.02
42.	MOL012266	Rivularin	37.94	0.37	16.25
43.	MOL000228	(2R)‐7‐hydroxy‐5‐methoxy‐2‐phenylchroman‐4‐one	55.23	0.20	17.02
44.	MOL002937	DIHYDROOROXYLIN	66.06	0.23	17.17
45.	MOL002925	5,7,2′,6’‐Tetrahydroxyflavone	37.01	0.24	18.00
46.	MOL002910	Carthamidin	41.15	0.24	15.81
47.	MOL002913	Dihydrobaicalin_qt	40.04	0.21	16.13
48.	MOL002914	Eriodyctiol (flavanone)	41.35	0.24	15.88
49.	MOL012245	5,7,4′‐trihydroxy‐6‐methoxyflavanone	36.63	0.27	16.12
50.	MOL012246	5,7,4′‐trihydroxy‐8‐methoxyflavanone	74.24	0.26	16.85

### 2.2 Potential Targets of the HYQB Capsule in the Treatment of AV

**FIGURE 2:**
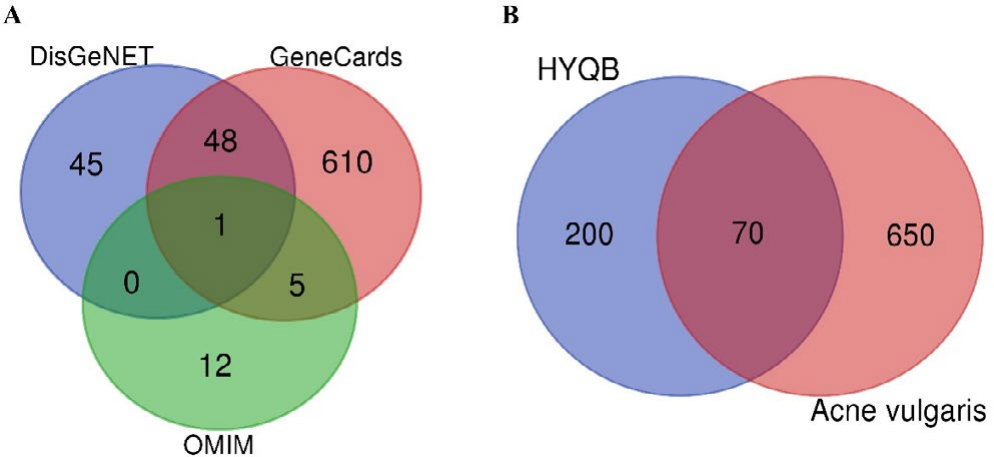
Targets associated with acne vulgaris and active ingredient targets of HYQB. (A) The diagram illustrating therapeutic targets for acne vulgaris. (B) Venn diagram of acne vulgaris targets and HYQB targets.

A total of 776 unique targets related to AV were collected from DisGeNET, Genecards, and OMIM databases (Figure 2A). After removing duplicates, 70 overlapping targets were recognized as possible therapeutic targets by comparing 270 potential targets related to HYQB capsules with AV‐related targets (Figure 2B).

### 2.3 Establishing and Analyzing the Network of the Herb‐Component‐Target

**FIGURE 3:**
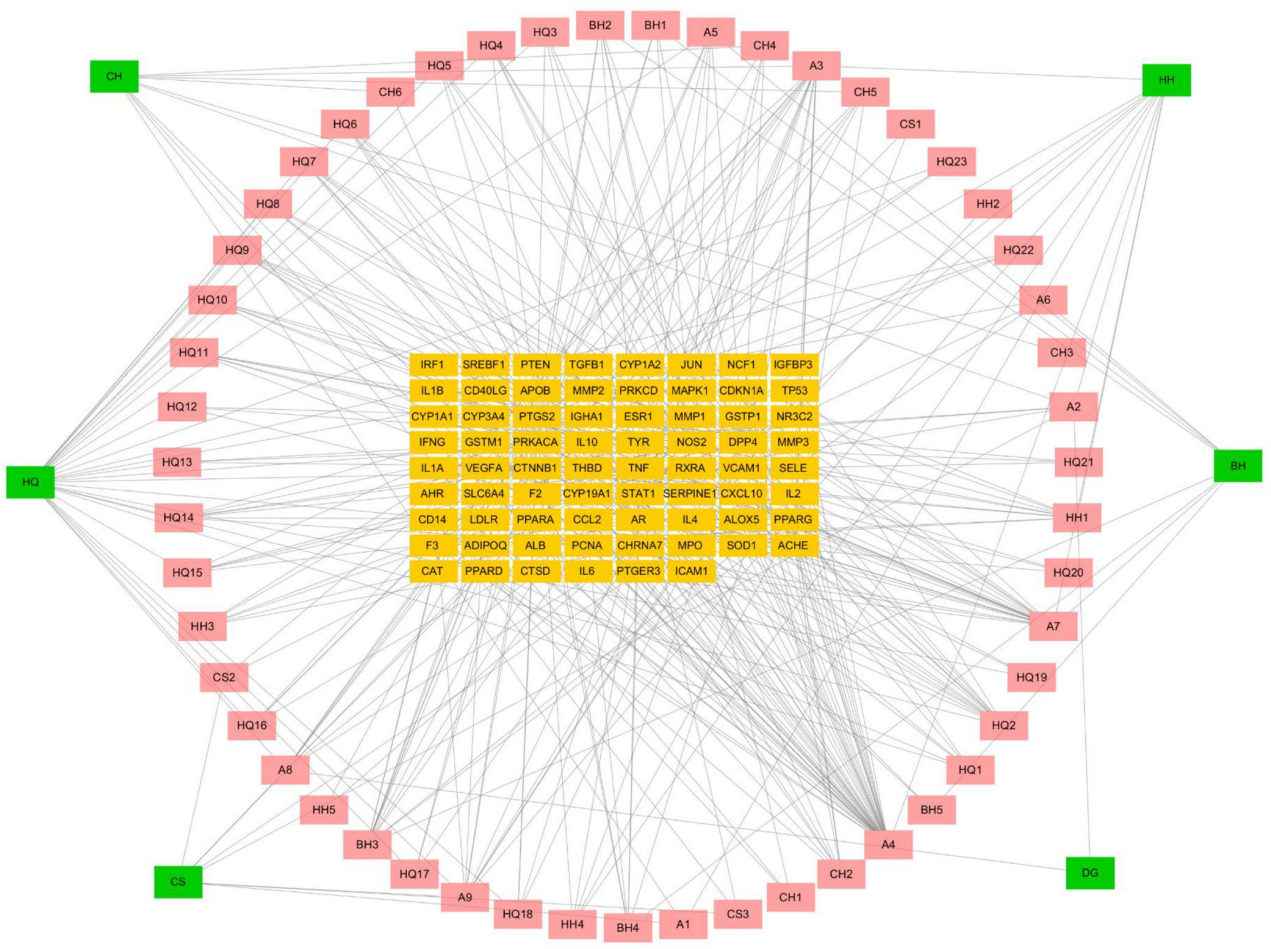
“Herb‐component‐target” network. The various herbs and active compounds of HYQB are symbolized by the green and pink rectangles. The yellow rectangles represent the core targets.

We built an interaction network in Cytoscape 3.7.2 to fully grasp the relationships between the candidate elements and potential targets of HYQB capsules, which include 6 natural remedies, 50 active ingredients, and 70 possible targets associated with acne vulgaris (Figure 3). This network includes 145 nodes (with compounds in pink, targets in yellow, and medicines in green) and 396 edges. Among these, quercetin, luteolin, kaempferol, and wogonin are the top four active components in terms of degree value. These substances have been shown to have a variety of medicinal effects, including anti‐inflammatory, antioxidant, and anti‐cancer properties [21, 22, 23, 24].

### 2.4 Exploration of a PPI Network for Possible Therapeutic Targets

**FIGURE 4:**
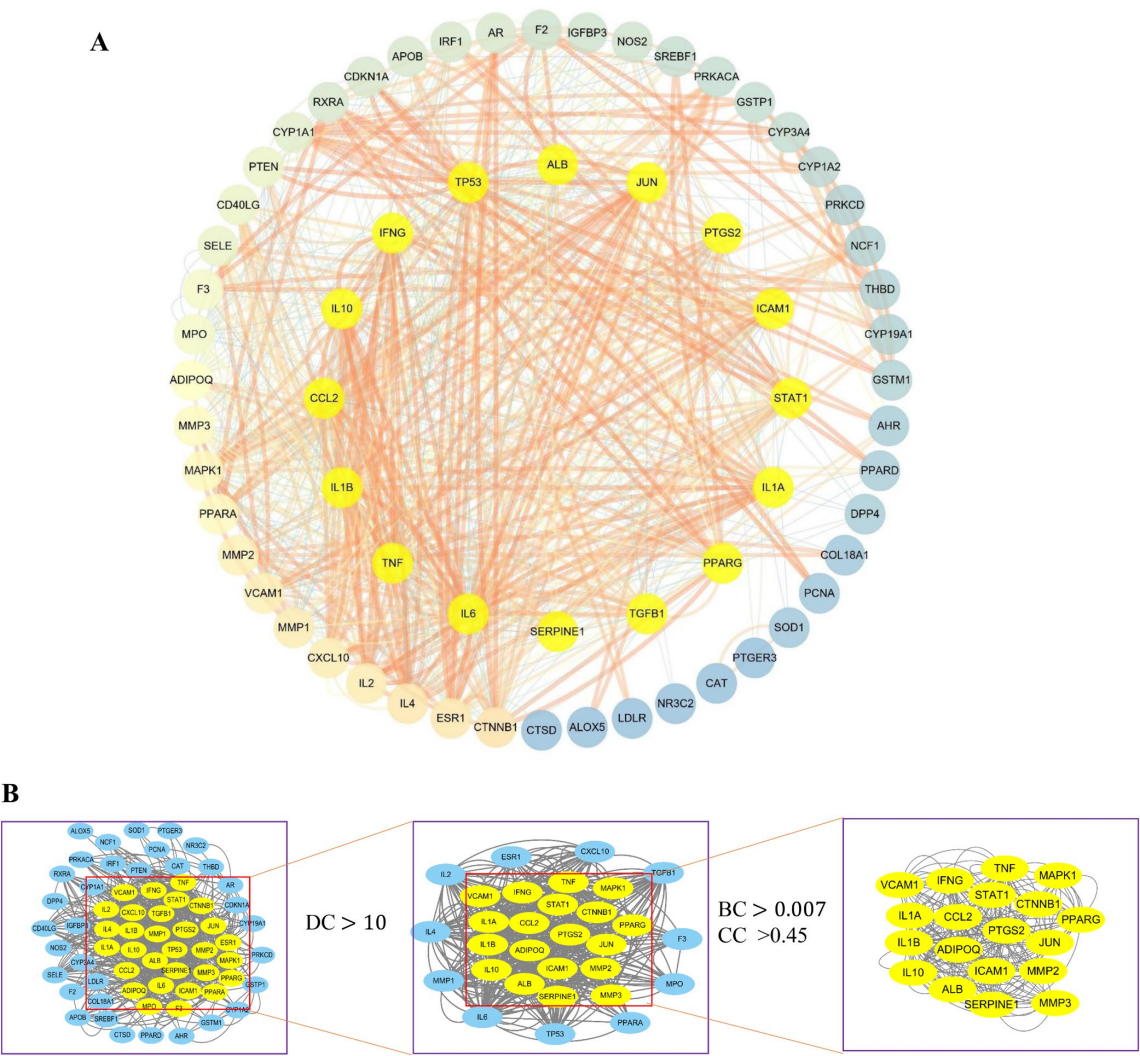
(A) The protein–protein interaction network of HYQB's targets used in treating AV. (B) Screening process for protein–protein interaction networks' topology.

In order to clarify how the HYQB capsule treats acne vulgaris, we created a PPI network by utilizing the STRING database and Cytoscape software (Figure 4A). The network consists of 64 nodes and 768 edges, with 6 proteins not involved in the network. Core targets were identified from the PPI network analysis using DC, BC, and CC values, and networks were built for both core and non‐core targets based on topological features (Figure 4B). Based on the topological parameter values, 19 targets from Table 2 were determined as the key targets for the HYQB capsule in the treatment of AV.

**TABLE 2:** Degree values of core targets for the HYQB capsule against AV.

No.	Targets name	BC	CC	Degree
1.	TNF	0.050	0.61	33
2.	IL1B	0.060	0.63	33
3.	CCL2	0.028	0.56	26
4.	IFNG	0.016	0.56	25
5.	IL10	0.017	0.54	25
6.	JUN	0.122	0.59	23
7.	PTGS2	0.075	0.56	23
8.	ICAM1	0.010	0.53	23
9.	ALB	0.086	0.58	23
10.	STAT1	0.028	0.54	22
11.	IL1A	0.010	0.53	22
12.	PPARG	0.041	0.55	20
13.	SERPINE1	0.028	0.49	19
14.	CTNNB1	0.027	0.55	18
15.	VCAM1	0.011	0.46	15
16.	MMP2	0.029	0.48	15
17.	MAPK1	0.022	0.51	13
18.	MMP3	0.014	0.46	13
19.	ADIPOQ	0.009	0.47	12

### 2.5 Performing GO and KEGG Pathway Enrichment Analysis

**FIGURE 5:**
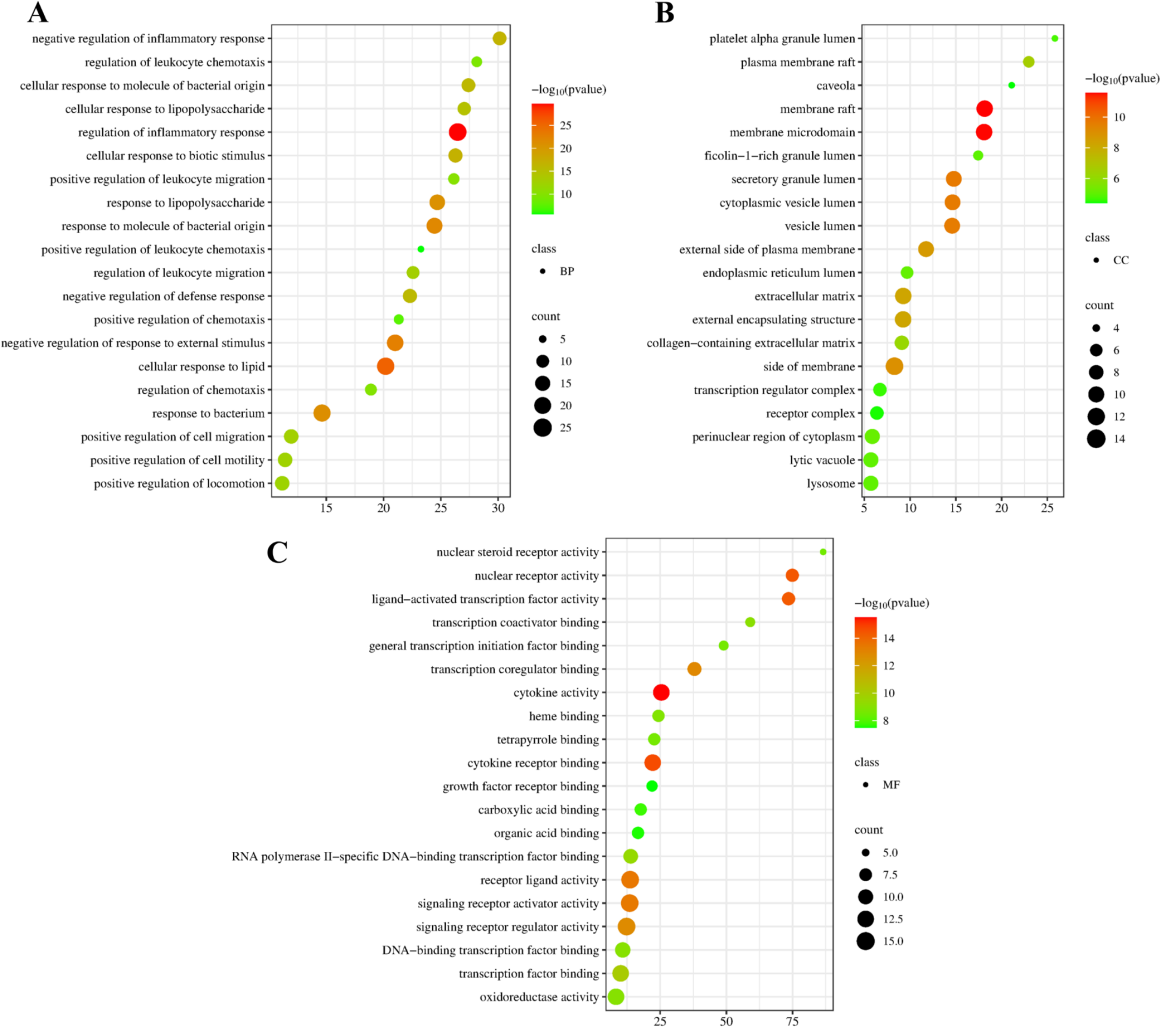
GO analysis results. (A) shows biological processes (BP); (B) shows cellular components (CC); (C) shows molecular functions (MF).

**FIGURE 6:**
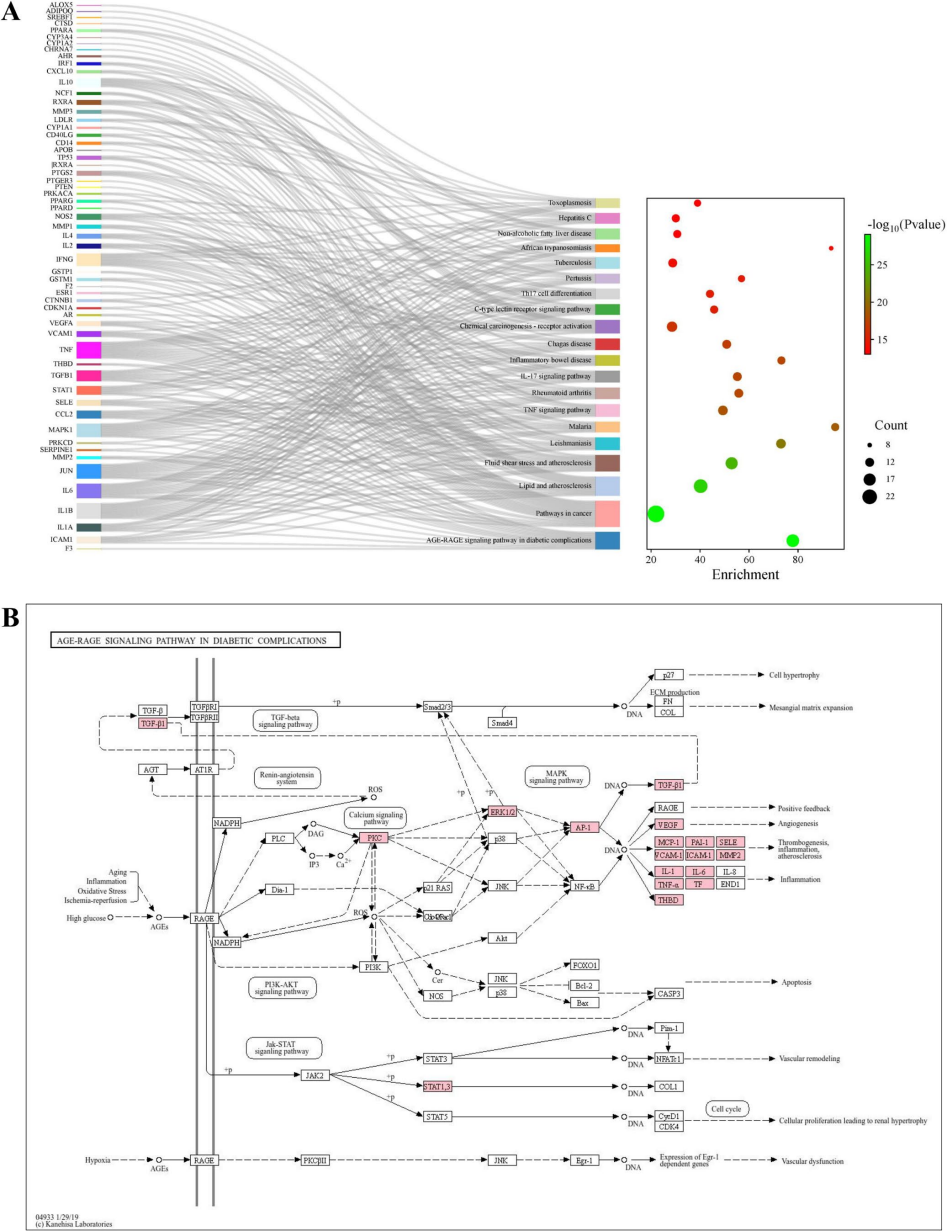
(A) Sankey diagram illustrates that the lines join the targets on the left and pathways on the right. (B) The AGE‐RAGE signaling pathway in complications related to diabetes.

Using the Metascape platform, the interrelationships and potential scientific implications of these core functional groups within biological networks were identified. Molecular function (MF) mainly consists of cytokine activity, binding to cytokine receptors, binding to transcription coregulators, and oxidoreductase activity. Cellular component (CC) primarily focuses on membrane raft, secretory granule lumen, cytoplasmic vesicle lumen, and plasma membrane raft. Biological process (BP) primarily involves the control of inflammatory reactions, cellular reactions to lipids, and the inhibition of responses to external stimuli (Figure 5A–C). The analysis of the enriched biological process ontology suggests that the anti‐AV properties of the HYQB capsule may be due to a combined, multi‐biological process synergistic impact that includes inflammation and oxidative stress. Enrichment analysis revealed significant KEGG pathways including the AGE‐RAGE signaling pathway in diabetic complications, TNF signaling pathway, IL‐17 signaling pathway, and Th17 cell differentiation with a *p*‐value < 0.05. The top 20 pathways are presented in Figure 6A. Furthermore, Figure 6B illustrates the closest association of the AGE‐RAGE signaling pathway in diabetic complications with AV.

### 2.6 Results of Molecular Docking

**FIGURE 7:**
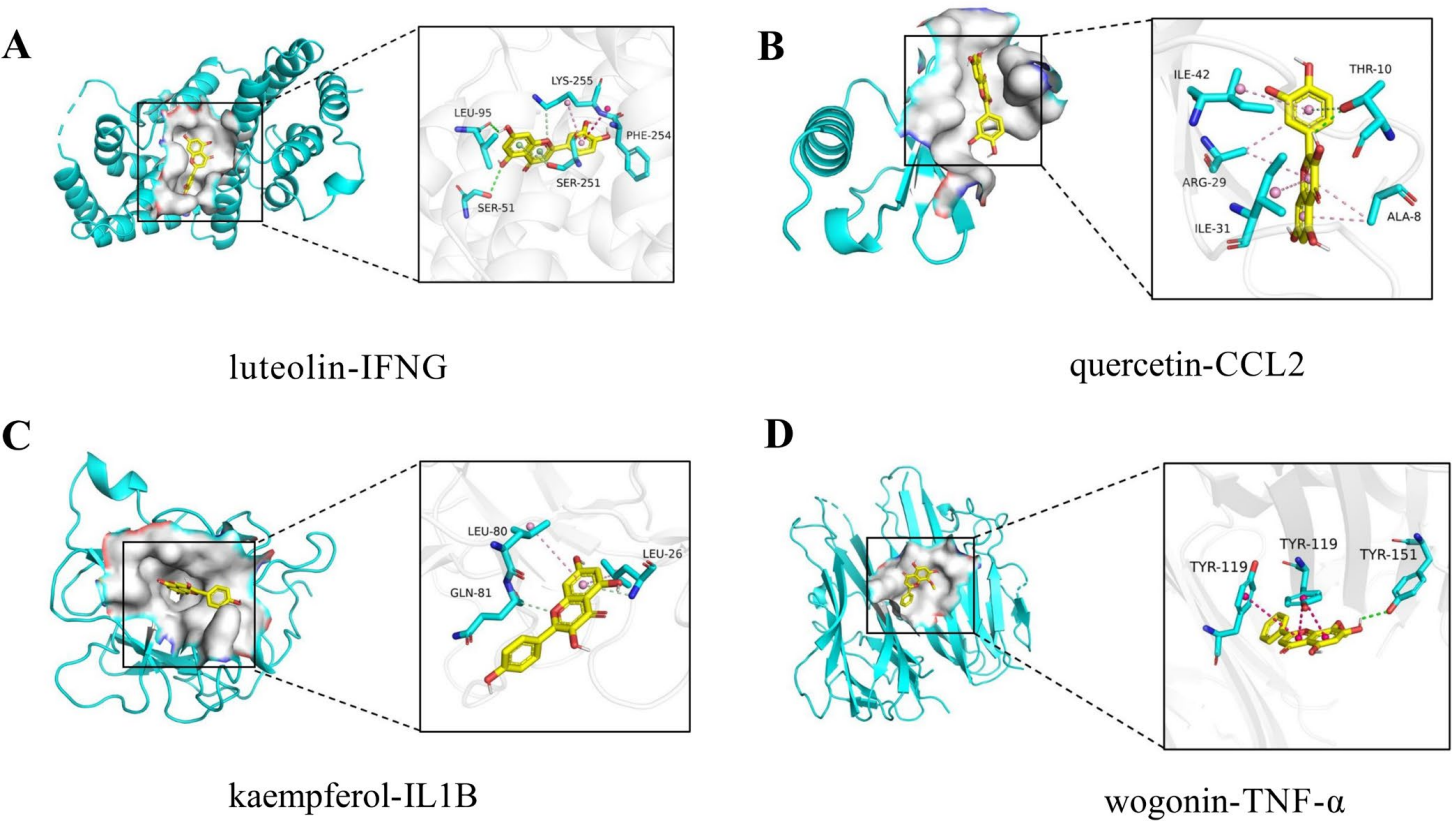
Molecular docking patterns between representative components and targets of YWD for treatment of acne vulgaris. (A) Luteolin‐IFNG. (B) Quercetin‐CCL2. (C) Kaempferol‐IL1B. (D) Wogonin‐TNF‐α.

The 4 active compounds screened from HYQB were docked with the first 4 core genes. The strength of molecular docking is determined by the binding energy, where a lower binding energy suggests a stronger bond between the ligand and receptor and a greater likelihood of activity [25]. A binding energy of −5.0 kJ/mol or less is considered the norm [26]. The results of the docking analysis revealed that all active ingredients exhibited an affinity below −5.0 kJ/mol for the specified targets. This further confirmed that the core active ingredients of HYQB have a good binding activity with the core targets for treating common acne, suggesting potential therapeutic effects. Notably, IFNG had the lowest binding energy among all docking groups, constituting the most stable conformation, which provides a basis for future applications of IFNG in the treatment of common acne. Moreover, luteolin showed the best docking effect with the core targets, having the lowest binding energy with all four core targets. Detailed docking results can be found in Table 3. Finally, we selected four representative molecular docking diagrams for display (Figure 7). Luteolin interacted with LEU95 and SER51 of IFNG through hydrogen bonds, quercetin bonded with THR10 of CCL2, and wogonin formed a hydrogen bond with TYR151 of TNF‐α. In addition, these small molecules all formed strong hydrophobic integration reactions with surrounding amino acid residues [27].

**TABLE 3:** Molecular docking predicts results of the key active ingredient and target protein.

Item	CCL2	IFNG	IL1B	TNF‐α
kaempferol	−5.7	−7.8	−6.8	−7.3
luteolin	−5.9	−7.9	−7.1	−7.8
quercetin	−5.9	−7.9	−7.0	−7.6
wogonin	−5.4	−7.5	−6.8	−7.3

*Note:* The binding free energy is kcal/mol.

## 3 Discussion

AV is a commonly occurring chronic inflammation of the hair follicles and sebaceous glands worldwide, and it is the most frequent skin condition in teenagers, affecting nearly all individuals in this age group. Manifestations of this condition may present in a rhythmic manner, occasionally with intense inflammation [28]. Traditional Chinese medicine attributes its development to imbalances in various organs like the lung, kidney, spleen, liver, heart, and large intestine as well as external influences like wind, cold, and dampness, which can result in the accumulation of phlegm, heat, and damp‐heat on the skin. While acne vulgaris may not be fatal, if left untreated or not properly managed, it can result in scarring and irritation and have a notable impact on a patient's mental health, potentially leading to depression. As such, it is crucial to seek out a treatment that is both safe and efficient.

HYQB, a pure Chinese medicine preparation, is utilized to address skin issues like chloasma and acne, particularly beneficial for individuals with stasis‐heat syndrome, sparking curiosity among the Chinese population. The main function of this formula is to balance the body by adjusting the interaction of unbalanced elements, addressing AV symptoms through actions like eliminating wind, reducing heat, improving blood flow, and resolving blockages. Radix Bupleuri, *Mentha spicata* L., *Scutellariae radix*, and *Radix paeoniae* possess properties that help clearing heat, cooling blood, drying dampness, and detoxification, while Angelicae Sinensis Radix and *Carthamus tinctorius* aid in improving blood flow and resolving blockages. Despite being available for an extended period of time, HYQB has shown considerable effectiveness in addressing AV. However, the specific components and how it works are still unknown, which is impeding the progress of the product. Network pharmacology is an innovative approach to designing and developing drugs that focuses on the idea of ‘disease‐multiple components‐multiple targets,’ aligning with the holistic perspective of traditional Chinese medicine. Hence, utilizing network pharmacology techniques can offer a novel method to investigate how HYQB treats AV.

AV is a complex condition with various histopathological characteristics, such as increased sebum production, follicular keratosis, growth of P. acne bacteria, and inflammation [29]. Seborrhea‐related sebaceous gland dysfunction is a key factor in the development of acne. Elevated sebum production, alterations in sebum fatty acid composition, hormonal imbalance, neuropeptide interactions, follicular hyperkeratosis, inflammation induction, and immune dysfunction (both congenital and acquired) are factors contributing to acne vulgaris [30]. By analyzing the target network of herbal compound therapy, we identified 50 active compounds as potential treatment components in the HYQB capsule for AV. Quercetin, acting as an antioxidant, can block the creation of pro‐inflammatory cytokines and has a suppressive impact on skin inflammation caused by acne propionic acid bacteria [31]. Luteolin can reduce the overactive sebaceous glands by moderating the decrease in androgen receptor (AR) expression [32] and trigger the API3K/NRF2/ARE system to produce anti‐inflammatory results [33]. Furthermore, it was noted that a compound can influence various targets. In vitro, wogonin can block the rise of IL‐1β production and PTGS2 expression caused by lipopolysaccharide [34], and in vivo, it can decrease the inflammatory reaction by lowering interleukin‐6 (IL‐6) and tumor necrosis factor α (TNF‐α), thus preventing the buildup of oxidative byproduct malondialdehyde (MDA) to ease oxidative stress and showing a potent anti‐lipid synthesis effect [35].

It is satisfying to observe that the majority of the core targets selected are linked to inflammatory infiltration and have the ability to control inflammatory reactions. Examples include CCL2 [36], STAT1 [37], and PPARG [38]. CCL2 not only enhances the movement of macrophages to the wound but also triggers macrophages to produce EGF, which in turn activates the growth of basal epidermal keratinocytes [39]. IL‐1 A, a cytokine, can lead to excessive keratinization of keratinocytes, causing the opening of sebaceous glands into the hair follicle to become narrower, thus impeding the flow of sebum. This sudden blockage can result in the development of acne. Loss of MMP3 can lead to delayed wound closure in skin repair [40], while decreased CTNNB1 expression can prevent excessive proliferation and abnormal differentiation of keratinocytes [41]. Additionally, the presence of intercellular adhesion molecule 1 (ICAM1) plays a role in cell migration, adhesion, angiogenesis, and vascular endothelial permeability [42]. AV is believed by Melnik BC [43] to be a consequence of metabolic syndrome affecting sebaceous glands, with reduced fat synthesis in skin tissue inhibiting acne progression. Conversely, short‐chain fatty acids (SCFAs) from acne propionic acid bacteria can induce lipid accumulation in keratinocytes and increase triglyceride production [44]. Fortunately, the key lipid metabolism target identified in this study, ADIPOQ, is primarily regulated by promoting fatty acid oxidation and inhibiting lipid synthesis [45]. Peroxisome proliferator‐activated receptor gamma (PPARG) controls the expression of genes involved in lipid and glucose metabolism, cell proliferation/differentiation, and inflammation pathways, thereby regulating sebum production and inflammation in SZ95 human sebaceous cells. Regulating PPARγ activity selectively could be a potential treatment approach for acne [46]. Quercetin, the primary ingredient of the HYQB capsule chosen through screening, is a hopeful natural remedy that enhances PPARG expression [47]. Overall, the active components in the HYQB capsule have demonstrated a diverse collaborative impact in treating AV.

The analysis of therapeutic targets using GO and KEGG enrichment suggested that the HYQB capsule may regulate inflammatory response, cell response to lipids, and oxygen levels by interacting with cytokines and receptors in membrane rafts, secretion granule cavities, and cytoplasmic vesicle cavities, as well as through oxidoreductase activity. It impacts several signaling pathways including the AGE‐RAGE pathway in diabetic complications, TNF, IL‐17, and Th17 cell differentiation and plays a role in multiple biological processes for the treatment of AV. In Figure 6, the AGE‐RAGE pathway in diabetic complications creates crosstalk effects in the network, controlling cell growth and specialization through various signaling pathways like PI3K‐AKT, Jak–STAT, MAPK, and TGF‐beta to trigger abnormal blood vessel growth in acne lesions. It can also increase inflammation by making blood vessels more permeable, worsening the immune response [48, 49, 50]. Propionibacterium acnes, a Gram‐positive bacterium linked to acne, can start inflammation, change T cell behavior, and contribute to acne development by promoting excessive secretion of T helper type 17 (Th17) cells and IL‐17 [51]. The study also analyzed key proteins and did molecular docking to improve target predictions. The top 5 hub targets are TNF, IL1B, CCL2, IFNG, and IL10. By examining the primary KEGG pathways, it is possible that the way HYQB treats AV is by suppressing the production of lipids in sebaceous glands and the differentiation of sebocytes. This may involve the anti‐inflammatory and antioxidant impacts of pathways like the AGE‐RAGE signaling pathway in diabetic complications and the TNF signaling pathway.

## 4 Conclusions

The study findings suggest that the HYQB capsule may have a broad impact on AV through various compounds and targets, potentially affecting pathways associated with immune inflammation response, lipid metabolism, abnormal angiogenesis, and cell proliferation. The HYQB capsule has been found to be beneficial in controlling the overproduction of sebum and differentiation of sebocytes at acne sites, in addition to its anti‐inflammatory effects. However, there are some limitations that need to be addressed. This study focused solely on the connections between the elements of the HYQB capsule and the targets associated with typical acne, neglecting the impact of various extraction techniques and herb dosages. Therefore, additional experimental confirmation is necessary to validate its healing properties.

## Ethics Statement

The authors have nothing to report.

## Conflicts of Interest

The authors declare no conflicts of interest.

## Supporting information


Table S1.

## Data Availability

The corresponding author can provide the data supporting the findings of this study upon a reasonable request.
