# Supplementary material for: The Therapeutic Mechanisms of Huayu Quban Capsule in Treating Acne Vulgaris Are Uncovered Through Network Pharmacology and Molecular Docking
Source: J Cosmet Dermatol. 2024 Nov 17;24(1):e16632. doi: 10.1111/jocd.16632 (PMC11743294; doi:10.1111/jocd.16632)
Supplement: Supplementary file 1 — Table S1. [file JOCD-24-e16632-s001.docx]

**Supplementary Table 1**  270 potential targets for the action of drug ingredients

| No | Target | No | Target | No | Target | No | Target | No | Target |
| --- | --- | --- | --- | --- | --- | --- | --- | --- | --- |
| 1 | LBP | 58 | CD14 | 115 | ALB | 172 | CYP2B1 | 229 | CYP3A4 |
| 2 | MMP10 | 59 | CTNNB1 | 116 | CA2 | 173 | PDE10A | 230 | TOP2 |
| 3 | MAP2 | 60 | OPRM1 | 117 | CALML1 | 174 | CHRNA2 | 231 | GABRA5 |
| 4 | CHRM4 | 61 | CYP2C9 | 118 | CDK7 | 175 | IGHA1 | 232 | CACNA2D1 |
| 5 | APOD | 62 | FABP5 | 119 | NOX5 | 176 | EGLN1 | 233 | KCNMA1 |
| 6 | TDRD7 | 63 | NFATC1 | 120 | ALOX12 | 177 | Cytc | 234 | MPO |
| 7 | FOSL2 | 64 | FOSL1 | 121 | HIF1A | 178 | SLC6A4 | 235 | DRD1 |
| 8 | FN1 | 65 | TEP1 | 122 | BBC3 | 179 | KDR | 236 | MET |
| 9 | ADCY2 | 66 | NUF2 | 123 | PTGES | 180 | XIAP | 237 | IL4 |
| 10 | TYR | 67 | MCL1 | 124 | CASP7 | 181 | ERBB2 | 238 | APP |
| 11 | MDM2 | 68 | CDK4 | 125 | CDKN1C | 182 | VEGFA | 239 | NFE2L2 |
| 12 | PRKCD | 69 | IL1B | 126 | PCNA | 183 | PRKCE | 240 | PKIA |
| 13 | SOAT1 | 70 | CES1 | 127 | ABAT | 184 | GOT1 | 241 | AKR1C1 |
| 14 | SOAT2 | 71 | ADIPOQ | 128 | ABCC1 | 185 | GSR | 242 | UGT1A1 |
| 15 | SREBF1 | 72 | HMGCR | 129 | PLB1 | 186 | APOB | 243 | MTTP |
| 16 | CAT | 73 | BAD | 130 | LDLR | 187 | MAPK3 | 244 | ampC |
| 17 | FASLG | 74 | FASN | 131 | PDE3A | 188 | RASA1 | 245 | CYP19A1 |
| 18 | IL1A | 75 | PLAT | 132 | PTEN | 189 | IFNG | 246 | CCNB1 |
| 19 | MGAM | 76 | THBD | 133 | E2F2 | 190 | E2F1 | 247 | SERPINE1 |
| 20 | IL2 | 77 | TGFB1 | 134 | HSPB1 | 191 | DUOX2 | 248 | RASSF1 |
| 21 | IL1B | 78 | GJA1 | 135 | PPARA | 192 | CLDN4 | 249 | RUNX2 |
| 22 | F3 | 79 | CHEK2 | 136 | PRKCB | 193 | SPP1 | 250 | BIRC5 |
| 23 | MYC | 80 | NR1I3 | 137 | IL8 | 194 | CHUK | 251 | PTGER3 |
| 24 | DCAF5 | 81 | CCL2 | 138 | MMP9 | 195 | CTSD | 252 | CXCL10 |
| 25 | CAV1 | 82 | MMP2 | 139 | AHR | 196 | ACP3 | 253 | ALOX5 |
| 26 | CRP | 83 | GSTP1 | 140 | CASP9 | 197 | EIF6 | 254 | CYP1B1 |
| 27 | CCL2 | 84 | HAS2 | 141 | CDKN1A | 198 | TP53 | 255 | PON1 |
| 28 | CYP3A4 | 85 | NR1I2 | 142 | IRF1 | 199 | ERBB3 | 256 | CDKN2A |
| 29 | VCAM1 | 86 | FOS | 143 | IL6 | 200 | BCL2L1 | 257 | CD40LG |
| 30 | SELE | 87 | RB1 | 144 | CCND1 | 201 | IGFBP3 | 258 | ICAM1 |
| 31 | IGF2 | 88 | IL10 | 145 | EGFR | 202 | CYP1A2 | 259 | CXCL11 |
| 32 | CYP1A1 | 89 | MMP3 | 146 | MAPK1 | 203 | ACACA | 260 | KCNH2 |
| 33 | HMOX1 | 90 | CRK2 | 147 | SLPI | 204 | HSPA5 | 261 | gyrB |
| 34 | STAT1 | 91 | AKR1C3 | 148 | PARP-1 | 205 | COL3A1 | 262 | RUNX1T1 |
| 35 | GSTM2 | 92 | MMP1 | 149 | CASP8 | 206 | HIF1A | 263 | GSTM1 |
| 36 | MAPK8 | 93 | TNF | 150 | INSR | 207 | NKX3-1 | 264 | PRKCA |
| 37 | BAX | 94 | NR1I3 | 151 | ODC1 | 208 | HK2 | 265 | NQO1 |
| 38 | CASP3 | 95 | PRXC1A | 152 | SOD1 | 209 | NRF2 | 266 | ABCG2 |
| 39 | NFKBIA | 96 | BCL2 | 153 | AHSA1 | 210 | RAF1 | 267 | PPP3CA |
| 40 | SLC2A4 | 97 | PSMD3 | 154 | JUN | 211 | DIO1 | 268 | NPEPPS |
| 41 | AKT1 | 98 | ELK1 | 155 | TOP1 | 212 | MPO | 269 | PCOLCE |
| 42 | TOP2B | 99 | TOP2A | 156 | GABRA2 | 213 | F2 | 270 | CYP101A1 |
| 43 | NCF1 | 100 | XDH | 157 | OLR1 | 214 | PIK3CG |  |  |
| 44 | CHEK1 | 101 | RELA | 158 | IKBKB | 215 | GRIA2 |  |  |
| 45 | PPARD | 102 | F7 | 159 | ACHE | 216 | NOS3 |  |  |
| 46 | PYGM | 103 | DPP4 | 160 | CCNA2 | 217 | CALM1 |  |  |
| 47 | AR | 104 | ESR1 | 161 | PRSS1 | 218 | PIM1 |  |  |
| 48 | PPARG | 105 | ESR2 | 162 | CDK2 | 219 | HSP90AA1 |  |  |
| 49 | NOS2 | 106 | ADRA1B | 163 | GSK3B | 220 | MAPK14 |  |  |
| 50 | GABRA3 | 107 | ADRA1A | 164 | GABRA1 | 221 | CHRNA7 |  |  |
| 51 | CHRM2 | 108 | LTA4H | 165 | MAOA | 222 | HTR2A |  |  |
| 52 | PTPN1 | 109 | ADRA2A | 166 | MAOB | 223 | SCN5A |  |  |
| 53 | F10 | 110 | NCOA1 | 167 | PLAU | 224 | ADRB1 |  |  |
| 54 | RXRA | 111 | IGHG1 | 168 | AKR1B1 | 225 | CHRM1 |  |  |
| 55 | NCOA2 | 112 | ADH1C | 169 | ADRB2 | 226 | CHRM3 |  |  |
| 56 | PTGS2 | 113 | NR3C2 | 170 | SLC6A3 | 227 | CTRB1 |  |  |
| 57 | PTGS1 | 114 | PGR | 171 | SLC6A2 | 228 | PRKACA |  |  |
